# Atrial fibrillation as a prognostic indicator of myocardial infarction and cardiovascular death: a systematic review and meta-analysis

**DOI:** 10.1038/s41598-017-03653-5

**Published:** 2017-06-13

**Authors:** Wenqi He, Yingjie Chu

**Affiliations:** Emergency department, Henan province People′s Hospital, Zhengzhou, Henan Province 450003 China

## Abstract

This study aimed to investigate whether atrial fibrillation (AF) predicts myocardial infarction (MI) or cardiovascular (CV) death. AF is a well-established risk factor for thrombotic stroke and all-cause mortality. PubMed, EmBase, and Cochrane Central were searched for articles comparing the incidence rates of MI, CV death, or CV events between AF and non-AF patients. Relative risk ratio (RR) was used as effect estimate. Crude and adjusted RRs were calculated. Data were pooled using a random-effects model. The meta-analysis included 27 studies. In the unadjusted analysis, AF patients had a nonsignificant trend toward a higher risk of MI compared with non-AF patients; however, a significant association was found. The crude data analysis showed that AF was associated with increased risk of CV death (*P* < 0.05) and CV events (*P* < 0.05). These associations remained significant after pooling data from adjusted models (CV death: RR = 1.95, 95% CI 1.51–2.51, *P* < 0.05; CV events: RR = 2.10, 95% CI 1.50–2.95, *P* < 0.05). These results showed that AF is an independent risk factor for MI, CV death, and CV events.

## Introduction

Atrial fibrillation (AF) is the most commonly encountered clinically significant cardiac arrhythmia. It represents a major public health problem with increasing prevalence in elderly people and significant association with poor outcomes^1, 2^. Approximately 2.7–6.1 million patients suffer from AF in the United States, a number expected to double by 2050^3^. AF is a well-established independent risk factor for stroke, and non-valvular AF increases the risk of stroke by fivefold^1, 4^. Antithrombotic medications, such as vitamin K antagonists (VKAs) and non-VKAs, are routinely recommended for AF patients to prevent stroke and systemic embolic events, while decreasing mortality^5, 6^.

Myocardial infarction (MI) has been recognized as a risk factor for new-onset AF since the Framingham Heart Study^7, 8^. Multiple studies demonstrated the unfavorable prognostic impact of AF in patients sustaining MI^9, 10^. The incidence of AF complicating acute MI is between 6% and 21%^9^. New-onset AF is associated with an increased risk of mortality even after adjusting for pivotal risk factors for AF^11, 12^. AF may lead to dismal prognosis in patients with MI through adverse hemodynamic effects, including loss of atrial contraction, high ventricular rates, loss of atrioventricular synchrony, and an irregular RR interval, leading to decreased cardiac output^13^.

Previous extensive studies have emphasized more on ischemic cerebrovascular rather than cardiovascular (CV) events, as the principal outcome in AF patients. A previous large cohort study suggested that AF is not strongly associated with ischemic heart disease^14^. Indeed, AF is associated with impaired coronary flow and diminished myocardial perfusion^15^. Numerous studies have illustrated the associations of AF with CV outcomes^3, 16, 17^. However, controversies exist because the annual rate of MI in AF patients is low, with drug intervention in biasing the size of effect estimate^18, 19^. For example,, warfarin, as a commonly prescribed drug for AF patients, may exert protective effects against MI^20^. Further, several studies suggested that the coexistence of atherosclerotic risk factors may contribute to increased risk of MI in AF^21, 22^. Also, concerns were raised regarding the increased risk of MI after use of non-VKAs, such as dabigatran^23–25^. Thus, a systematic review and meta-analysis was conducted to compare cardiovascular outcomes between AF and non-AF patients.

## Methods

### Data Sources, Search Strategy, and Selection Criteria

The present systematic review and meta-analysis adhered to the Preferred Reporting Items for Systematic Reviews and Meta-Analyses guidelines^26^. PubMed, EmBase, and Cochrane Central were searched from inception to June 2016. The following key words and medical terms were used in the literature search: “atrial fibrillation” AND (“acute coronary syndrome” OR “myocardial infarction” OR “coronary heart disease” OR “myocardial ischemia” OR “angina” OR “cardiac death” OR “cardiac event” OR “cardiac mortality” OR “cardiac events” OR cardiovascular) AND (random* OR trial OR cohort OR retrospective* OR database OR population-based OR “population based” OR prospective* OR follow-up OR follow up OR registry OR community-based). The search was limited to the English language. The references of included studies were manually screened for potentially eligible studies.

For inclusion in the meta-analysis, studies had to meet the following criteria: adult patient evaluation; availability of extractable data for MI, CV death, or CV events in individuals with AF. Both retrospective and prospective studies were selected. Studies that enrolled AF patients during the postoperative phase were excluded. The primary outcome of interest was MI, and secondary outcomes included CV death and CV events. The latest American College of Cardiology/American Heart Association guidelines suggested that CV death might be attributed to MI, sudden cardiac death (SCD), heart failure (HF), stroke, cardiovascular procedure, or vascular hemorrhage^27^. Among the included studies, the “cardiovascular” categories might vary widely. However, studies focusing only on the outcome of SCD, for which ventricular fibrillation was the predominant cause, were not considered. Studies including only AF patients after MI were excluded as well, considering that AF might be complicated by MI, leading to a bias in exploring their association.

### Data extraction

Two researchers respectively screened the titles and abstracts, and excluded studies not meeting the inclusion criteria. Potentially eligible full-text articles were subsequently reviewed. In case of disagreement, consensus was achieved through consultation with the corresponding author. The following information was extracted: author, year, study design, region, number of patients, gender, population, AF diagnosis method, proportion of patients with prior MI, CHADS2 score, antithrombotic medications, cardiovascular outcomes, degree of adjustment, and follow-up. Crude and adjusted RRs were both directly extracted or indirectly calculated. The adjustment degree was categorized as “+” for age and/or sex only; “++” for those further adjusting for less than five standard vascular risk factors (i.e., blood pressure/hypertension, smoking, drinking, and body mass index); and “+++” for those further adjusting five or more risk factors, including unconventional or socioeconomic factors. The quality of the included studies was appraised by the Newcastle–Ottawa Scale (NOS)^28^. This scoring system mainly incorporated four aspects, including selection, comparability, exposure, and outcomes, with a total score ranging from 0 to 7 points: 0–2 points, low quality; 3–5 points, medium quality; more than 6 points, high quality.

### Statistical analysis

Relative risk RATIO (RR) with associated 95% confidence interval (CI) was considered the effect estimate for binary outcomes. Hazard ratio was considered to be equivalent to RR in cohort studies. Given the low incidence of MI in AF patients, odds ratios (ORs) could be assumed to be accurate estimates of RRs. Crude and adjusted RRs were pooled separately. When adjusted RRs were presented by multiple multivariate models, the most adjusted one was selected. The meta-analysis was performed using a random-effects model^29^. Heterogeneity was assessed by the *I* ^2^ statistics^30^. Publication bias was assessed visually using funnel plots, and statistically by Begg’s and Egger’s tests^31, 32^. Also, sensitivity analysis was performed by excluding the included studies one by one. A stratified analysis was performed to assess the effects of publication year (2010 or after vs before 2010), study design (prospective cohort vs retrospective cohort), region (North America, Europe, or Asia), sample size (≥10000 vs <10000), mean age (60 years or older vs <60 years), female proportion (≥50% vs <50%), previous MI (≥20% vs <20%), adjustment degree (+,++, or+++), and follow-up duration (≥5 years vs <5 years). A meta-regression was conducted to investigate the impact of publication year, sample size, mean age, women proportion (%), previous MI (%) and follow-up duration on data heterogeneity. All analyses were conducted using the Stata software (version 12.0, Stata Corporation, TX, USA). All *P* values were two sided, with a significance level of 0.05.

## Results

**Figure 1:**
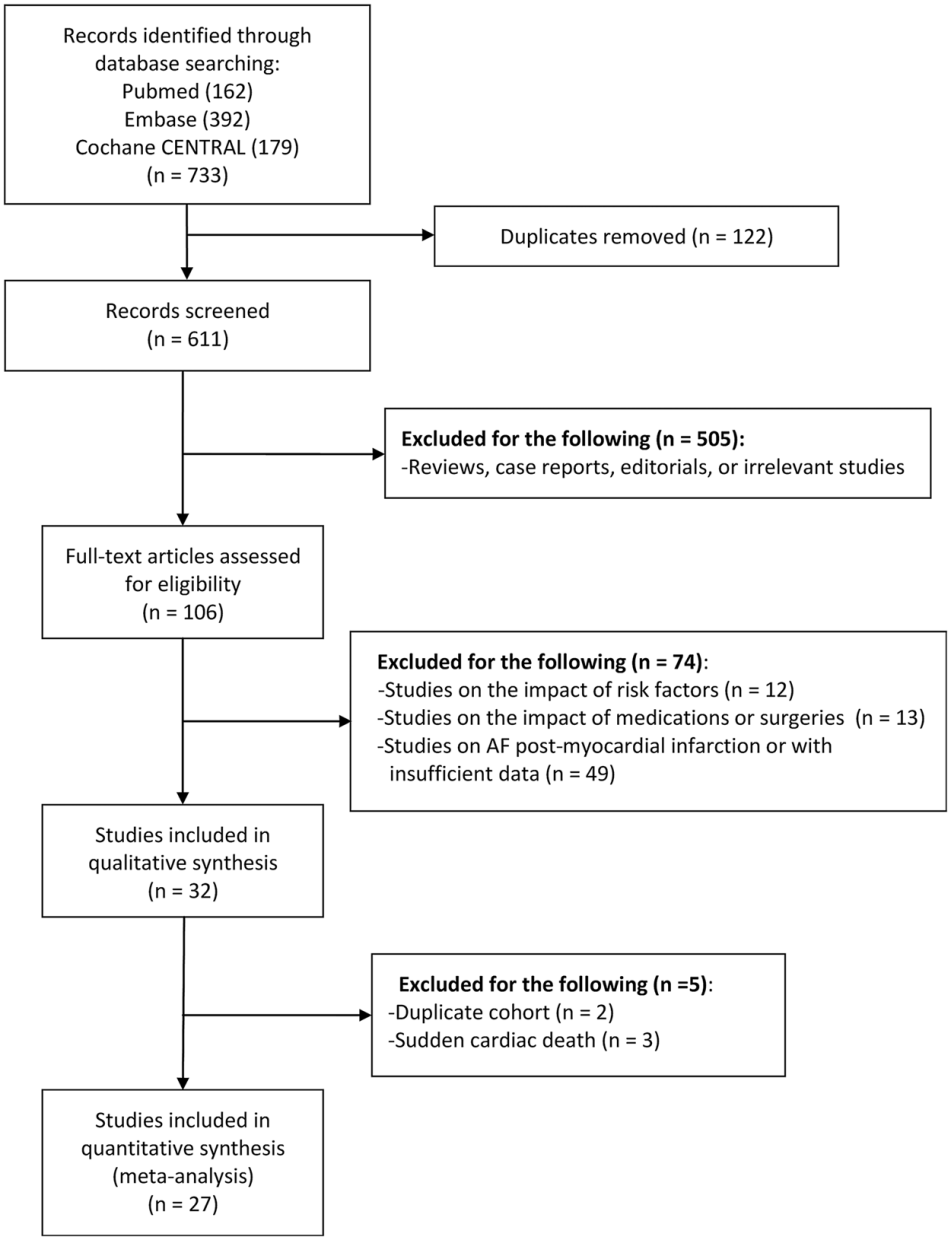
Flow diagram showing the study selection process.

A total of 733 records from the initial search were identified, including 162 from PubMed, 392 from EmBase, and 179 from Cochrane Central. After discarding 122 duplicates and 505 irrelevant studies, 106 full-text articles were assessed. Further, studies that enrolled single-arm AF patients, included AF patients after ischemic coronary disease, explored the impact of risk factors, or focused on medications/surgeries were excluded. Thirty-two studies were included in a qualitative analysis. Then, two studies of duplicate cohorts and three articles assessing SCD were excluded. The study selection process is displayed in Fig. 1.

### Characteristics of the included studies

Overall, 27 studies fulfilled the inclusion criteria and were included in the final meta-analysis^3, 8, 16, 33–56^. Table 1 summarizes the baseline characteristics of these studies. They included 20 and 7 prospective and retrospective studies, respectively. The number of enrolled patients ranged from 590 to 704,225, with a total of 1,324,037 patients. Eleven studies were performed in North America, 5 in Asia, and 9 in Europe. Two were international multicenter studies. The median or mean follow-up duration ranged from 14 days to 44 years. In quality assessment using the NOS scale (Supplementary Table S1), 16 (59.3%) studies achieved high-quality scores of 6–7. The least satisfying items included the representativeness of patients (14/27), adequacy of follow-up (13/27), and control of confounding factors (18/27).

**Table 1:** Characteristics of included studies.

Author (year)	Study design	Region	Patients (*n*)	Population	AF diagnosis	Age (year)	Female (%)	Previous MI (%)	CHADS 2 scores	AT	Degree of adjustment	CV outcomes	Follow-up
Kannel (1982)	Prospective	USA	590	Framingham cohort	ECG	48	55.2	6.9	NA	NA	None	CV death	22 years
Lake (1989)	Prospective	Australia	1,770	Community-based	Record	>60	48	11	NA	NA	+++	CV death	17 years
Krahn (1995)	Prospective	Canada	3,983	Healthy pilots	ECG (85%)	31	0	22.3	NA	NA	+++	CV death, MI	44 years
Aronow (1995)	Prospective	USA	1,359	Heart disease	ECG	81	70.2	47.1	NA	NA	++	Coronary events	42 months
Kaarisalo (1997)	Prospective	Finland	6,912	First ischemic stroke	ECG (>80%)	64	44	NA	NA	NA	None	CV death	1 year
Benjamin (1998)	Prospective	USA	1,863	Framingham cohort	ECG	75	52.3	13.6	NA	NA	None	CV death	40 years
Dries (1998)	Retrospective	USA	6,517	Heart failure	ECG	60	14	74.5	NA	10.7	None	MI	33.4 months
Saxena (2001)	Prospective	International	18,451	Acute stroke	Record	72	46.6	NA	NA	NA	None	Coronary death	14 days
Friberg (2004)	Prospective	Denmark	29,310	Community-based	ECG	58	55.8	2.6	NA	4.4	+++	CV death	4.7 years
Dhamoon (2007)	Prospective	USA	655	First ischemic stroke	Record	70	55.4	16.2	NA	NA	+++	CV events	4 years
Goto (2008)	Prospective	International	63,589	Atherothrombotic disease	Record	68	36	31	0–6	86.3	None	CV death, nonfatal MI	1 year
Ruigómez (2009)	Retrospective	UK	9,057	Community-based	Record	40–89	53.4	NA	NA	NA	+++	Coronary events	6 years
Haywood (2009)	Prospective	USA	39,056	Hypertension	ECG (92.1%)	≥55	45.9	25.7	NA	36.6	None	Cardiac events	4.9 years
Bouzas-Mosquera (2010)	Retrospective	Spain	17,100	Patients with known or suspected CAD	ECG	64	41	17.3	NA	NA	+++	MI	6.5 years
Winkel (2010)	Prospective	Europe	3,655	PAD	Record	68	24.8	25.8	NA	NA	+++	CV events, CV death	2 years
Conen (2011)	Prospective	USA	34,722	Community-based	Record	53	100	NA	0–5	NA	+++	CV death	15.4 years
Aguilar (2012)	Prospective	Spain	3,848	PAD, CAD, or CVD	Record	58	25.8	37.3	0–6	7.6	None	MI	16 months
Chao (2014)	Retrospective	Taiwan	24,228	Healthy community-based	Record	47	40.1	NA	0–1	NA	+++	AMI	5.7 years
Martinez (2014)	Retrospective	Austrilia	30,260	Asymptomatic AF	Record	71	38.4	4.7	1.1	NA	None	MI	3 years
Soliman (2014)	Prospective	USA	23,928	Community-based	ECG or record	64	58.2	0	40.4	NA	+++	MI	6.9 years
Albayrak (2015)	Prospective	Turkey	2,230	Community-based	ECG	50	63.9	NA	NA	NA	+++	CV events	3 years
Vermond (2015)	Prospective	Netherlands	8,265	Community-based	ECG	49	50.2	3	NA	NA	Adjusted	CV events, cardiac events	9.7 years
Soliman (2015)	Prospective	USA	14,462	Community-based	ECG or record	54	56	0	45.7	NA	+++	MI	21.6 years
Li (2015)	Retrospective	Taiwan	704,225	Community-based	Record	>18	44.4	NA	NA	NA	+++	CV events	4 years
Parisi (2015)	Prospective	UK	256,710	Psoriasis	Record	48	56.3	NA	NA	NA	+++	CV events	5.2 years
Shih (2016)	Retrospective	Taiwan	12,988	Hemodialysis	Record	69	53.3	14	0–9	8.4%	+++	CV death, MI	3.2 years
O’Neal (2016)	Prospective	USA	4304	No CV disease	ECG or record	>65	61	NA	NA	1.4	+++	CHD, MI	11 years

AT, antithrombotic; ACM, all-cause mortality; CHD, coronary heart disease; CV, cardiovascular; CVD, cardiovascular disease; HF, heart failure; MI, myocardial infarction; NFMI, nonfatal MI; PAD, peripheral arterial disease.

### Myocardial infarction

**Figure 2:**
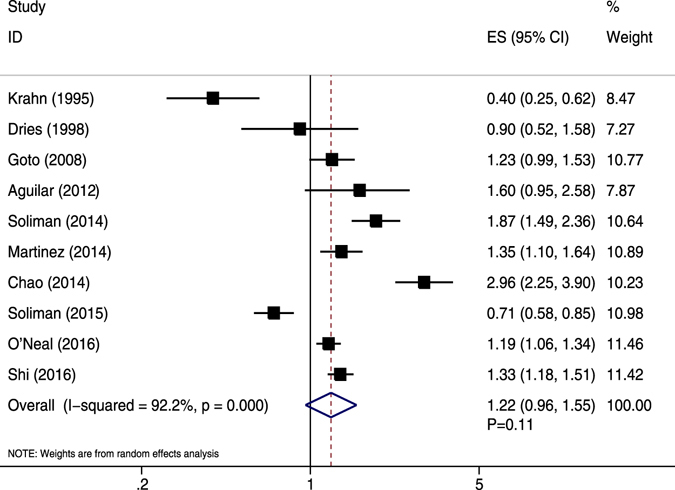
Forest plot showing the comparison between AF and non-AF patients in the pooled analysis of crude relative risk for myocardial infarction.

Crude unadjusted data relating to MI were presented in 11 studies^3, 16, 35, 38, 42, 46, 48–51, 56^. Two studies investigated the same cohort (REACH registry)^42, 46^, and the one with the largest sample size was selected^42^. Thus, 10 studies were included in the current meta-analysis. Dries *et al*. showed two sub-cohorts that were aggregated using fixed- and random-effects models^38^. Compared with non-AF patients, AF patients had an insignificant trend toward a higher risk of MI (RR = 1.22, 95% CI 0.96–1.55, *P* = 0.11), with high heterogeneity (*I* ^2^ = 92.2%, *P* < 0.05) (Fig. 2). Sensitivity analysis by excluding the included studies one by one showed no substantial alteration.

The meta-regression showed a significant impact of follow-up duration on the overall effect for unadjusted MI (*P* = 0.010). However, publication year, sample size, mean age, female proportion, and previous MI were not significant contributors of the association between AF and unadjusted MI (Table 2). The findings of subgroup analyses are presented in Table 3. Interestingly, AF was significantly correlated with increased risk of unadjusted MI in studies published after 2010; conducted in Asia; with retrospective design, sample size ≥ 10000, mean age ≥60.0 years, and follow-up duration <5.0 years. No other significant associations of AF with unadjusted MI were detected (Table 3).

**Table 2:** Meta-regression findings.

Outcomes	Publication year	Sample size	Age	Female (%)	Previous MI (%)	Follow-up duration
Unadjusted MI	0.058	0.419	0.243	0.188	0.774	**0.010**
Adjusted MI	0.910	0.378	0.693	0.595	**0.020**	0.667
Unadjusted CV death	0.473	0.507	0.561	0.418	0.535	0.655
Adjusted CV death	0.719	**0.040**	0.640	0.206	0.067	0.350
Unadjusted CV events	0.546	**0.009**	0.233	0.326	0.243	0.299
Adjusted CV events	0.913	0.082	0.390	0.748	0.581	0.728

**Table 3:** Subgroup analyses of crude relative risk for myocardial infarction and adjusted relative risk for myocardial infarction.

Outcomes	Group	RR and 95%CI	P value	Heterogeneity (%)	P value for heterogeneity	P value between subgroups
**Crude relative risk for myocardial infarction**	Publication year					
	2010 or after	1.43 (1.09–1.87)	0.011	93.1	<0.001	0.006
	Before 2010	0.77 (0.37–1.60)	0.482	90.3	<0.001	
	Study design					
	Prospective	1.05 (0.75–1.47)	0.789	92.5	<0.001	<0.001
	Retrospective	1.53 (1.05–2.23)	0.027	90.3	<0.001	
	Region					
	North America	0.92 (0.60–1.39)	0.68	93.7	<0.001	<0.001
	Europe	1.60 (0.97–2.64)	0.065	—	—	
	Asia	1.72 (1.13–2.61)	0.011	92.9	<0.001	
	International	1.23 (0.99–1.53)	0.061	—	—	
	Sample size					
	10000 or greater	1.42 (1.02–1.97)	0.036	94	<0.001	0.016
	<10000	0.91 (0.53–1.57)	0.743	87.9	<0.001	
	Mean age					
	60 or older	1.33 (1.16–1.51)	<0.001	63.9	0.017	0.005
	<60	1.07 (0.45–2.59)	0.872	96.8	<0.001	
	Women proportion					
	≥50%	1.20 (0.88–1.63)	0.246	93.6	<0.001	0.051
	<50%	1.21 (0.77–1.91)	0.41	92.2	<0.001	
	Previous myocardial infarction					
	≥20%	0.92 (0.52–1.63)	0.774	87.4	<0.001	0.025
	<20%	1.24 (0.86–1.77)	0.248	93.7	<0.001	
	Adjustment degree					
	+++	1.19 (0.83–1.70)	0.347	95.5	<0.001	0.631
	None	1.28 (1.12–1.47)	<0.001	0	0.455	
	Follow-up duration					
	≥5 years	1.15 (0.70–1.90)	0.586	96.4	<0.001	0.148
	<5 years	1.31 (1.19–1.43)	<0.001	0	0.599	
**Adjusted relative risk for myocardial infarction**	Publication year					
	2010 or after	1.45 (1.07–1.99)	0.018	91.3	<0.001	0.241
	Before 2010	1.02 (0.66–1.58)	0.93	—	—	
	Study design					
	Prospective	1.48 (1.25–1.74)	<0.001	35.4	0.2	0.013
	Retrospective	1.34 (0.66–2.72)	0.414	95.9	<0.001	
	Region					
	North America	1.48 (1.25–1.74)	<0.001	35.4	0.2	0.002
	Europe	0.77 (0.53–1.11)	0.166	—	—	
	Asia	1.75 (0.65–4.73)	0.272	97.6	<0.001	
	Sample size					
	10000 or greater	1.42 (0.84–2.41)	0.188	94.3	<0.001	0.106
	<10000	1.41 (1.15–1.73)	0.001	46.6	0.154	
	Mean age					
	60 or older	1.20 (0.92–1.56)	0.187	81.5	0.001	<0.001
	<60	1.73 (1.02–2.93)	0.04	89.3	<0.001	
	Women proportion					
	≥50%	1.40 (1.10–1.78)	0.007	83	0.001	0.036
	<50%	1.33 (0.55–3.24)	0.528	94.6	<0.001	
	Previous myocardial infarction					
	≥20%	1.02 (0.66–1.58)	0.93	—	—	<0.001
	<20%	1.24 (0.91–1.70)	0.18	86.6	<0.001	
	Adjustment degree					
	+++	1.39 (1.05–1.85)	0.022	89.8	<0.001	—
	++	—	—	—	—	
	Follow-up duration					
	≥5 years	1.47 (1.07–2.02)	0.018	87.1	<0.001	<0.001
	<5 years	1.06 (0.93–1.20)	0.366	—	—	

**Figure 3:**
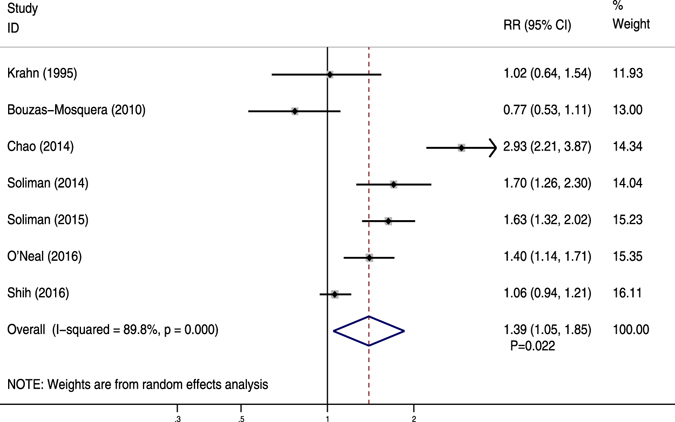
Forest plot showing the comparison between AF and non-AF patients in the pooled analysis of adjusted relative risk for myocardial infarction.

Adjusted data regarding MI were described in seven studies^3, 16, 35, 45, 49, 51, 56^. All studies had a sufficient degree of adjustment (+++). AF was significantly associated with a reduction in the risk of MI (RR = 1.39, 95% CI 1.05–1.85, *P* < 0.05) (Fig. 3). A high level of heterogeneity was present (*I* ^2^ = 89.8%, *P* < 0.05). No substantial change in the overall effect was shown after exclusion of any individual study.

The meta-regression showed previous MI contributed to the association of AF with adjusted MI (*P* = 0.020). Publication year, sample size, mean age, female proportion, and follow-up duration did not appear to affect meta-regression data (Table 2). The results of stratified analyses showed that the association remained significant for studies published after 2010; conducted in North America; with prospective design, sample size <10000, mean age <60.0 years, female proportion≥50%, and follow-up duration≥5.0 years (Table 3).

### CV death

**Figure 4:**
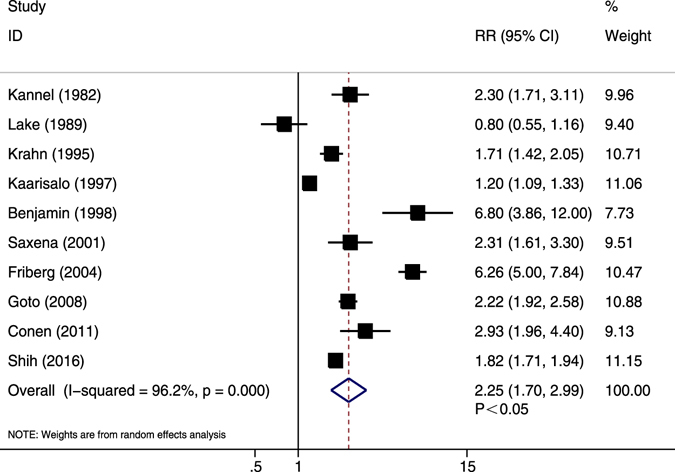
Forest plot showing the comparison between AF and non-AF patients in the pooled analysis of crude relative risk for cardiovascular mortality.

Ten studies displayed crude data^8, 33, 35–37, 39, 40, 42, 47, 56^. Except for one retrospective study^56^, most studies were prospectively designed. Compared with non-AF patients, AF patients had significantly increased risk of CV death (RR = 2.25, 95% CI 1.70–3.00, *P* < 0.05) (Fig. 4). High heterogeneity was revealed (*I* ^2^ = 96.2%, *P* < 0.05). Sensitivity analysis by excluding any single study did not alter the overall effect. Meta-regression suggested that publication year, sample size, mean age, female proportion, previous MI, and follow-up duration did not account for data heterogeneity (Table 2). In subgroup analysis (Supplementary Table S2), the results turned to be insignificant for two Asian studies (*P* = 0.612) and two European trials (*P* = 0.224).

**Figure 5:**
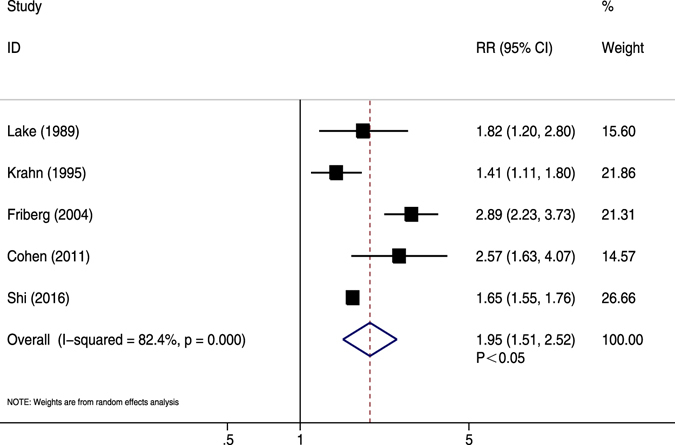
Forest plot showing the comparison between AF and non-AF patients in the pooled analysis of adjusted relative risk for cardiovascular mortality.

Adjusted data were available from five studies^33, 35, 40, 47, 56^. The pooled data revealed that AF patients were at a higher risk of CV death compared with non-AF patients (RR = 1.95, 95% CI 1.51–2.51, *P* < 0.05; *I* ^2^ = 82.3%, *P* < 0.05) (Fig. 5). After excluding the only retrospective study^56^, the significant trend was not markedly changed (RR = 2.08, *P* < 0.05). The meta-regression showed sample size contributed to the association of AF with adjusted MI (*P* = 0.040). Publication year, mean age, female proportion, previous MI and follow-up duration did not appear to affect meta-regression data (Table 2). These findings in stratified analyses were consistent with overall analysis for adjusted CV death (Supplementary Table S2).

### CV events

**Figure 6:**
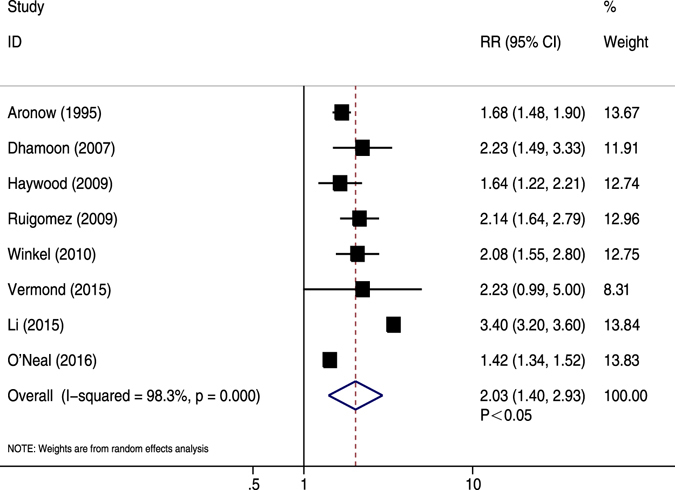
Forest plot showing the comparison between AF and non-AF patients in the pooled analysis of crude relative risk for cardiovascular events.

Crude data were shown in eight studies^16, 34, 41, 43, 44, 46, 53, 55^. AF patients had substantially higher risk of CV events compared with non-AF patients (RR = 2.03, 95% CI 1.40–2.93, *P* < 0.05). A high heterogeneity was demonstrated (*I* ^2^ = 98.3%, *P* < 0.05) (Fig. 6). After sequential exclusion of each study from pooled analyses, the conclusion was not affected. The finding of meta-regression showed sample size contributed to the association of AF and unadjusted CV events (*P* = 0.009) (Table 2). Subgroup analyses showed AF was not associated with the risk of unadjusted CV events with mean age <60 years (Supplementary Table S3).

**Figure 7:**
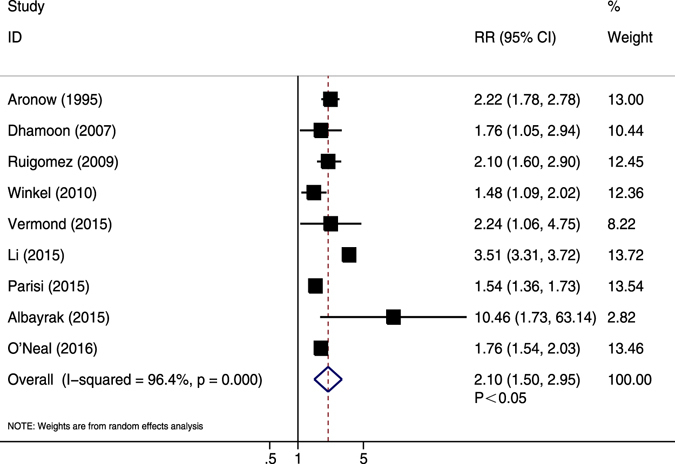
Forest plot showing the comparison between AF and non-AF patients in the pooled analysis of adjusted relative risk for cardiovascular events.

Nine studies showed adjusted data^16, 34, 41, 44, 46, 53–55^. Except for one study with an adjustment degree of “++”^34^, most studies showed a sufficient degree of adjustment (+++). The pooled results demonstrated that AF patients had significantly higher risk of CV events compared with those without AF (RR = 2.10, 95% CI 1.50–2.95, *P* < 0.05; *I* ^2^ = 96.4%, *P* < 0.05) (Fig. 7). Sensitivity analysis did not suggest a substantial effect for any single study. Publication year, sample size, mean age, female proportion, previous MI and follow-up duration did not appear to affect the association of AF with adjusted CV events (Table 2). The significant trend was changed with female proportion <50% (Supplementary Table S3).

### Publication bias

**Figure 8:**
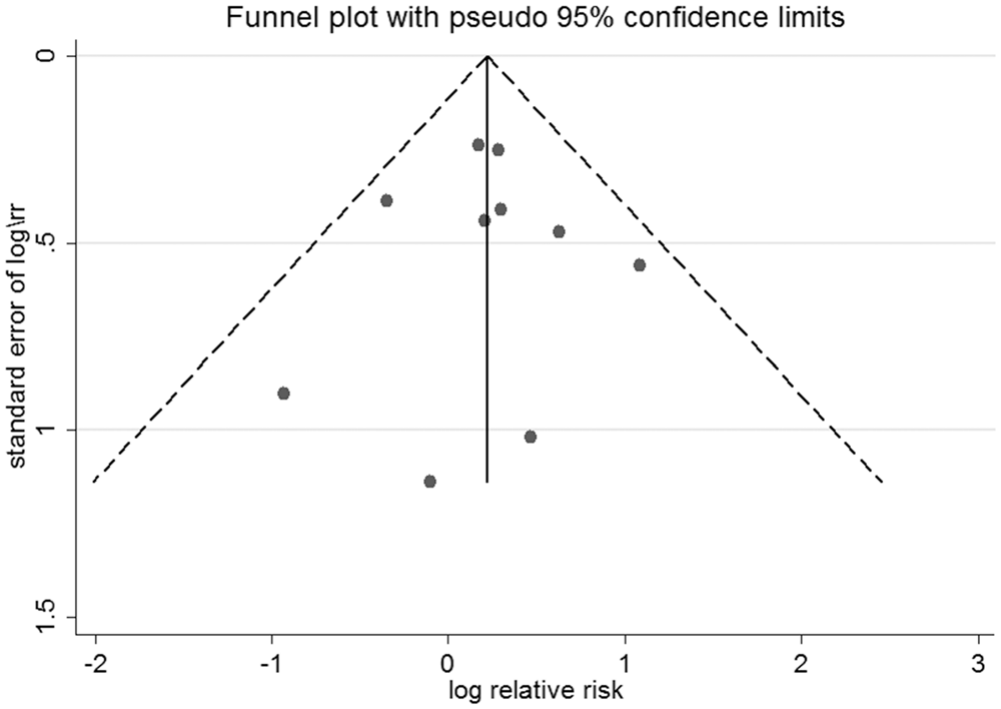
Funnel plot of studies assessing the myocardial infarction outcome.

**Figure 9:**
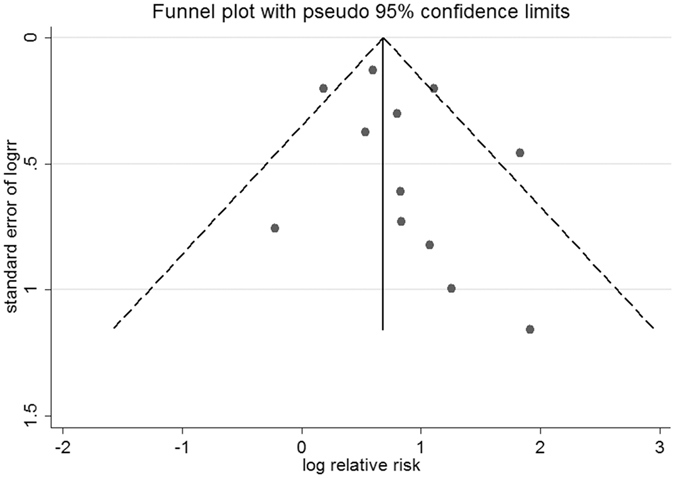
Funnel plot of studies assessing the CV death outcome.

For studies assessing MI outcomes, no publication bias was revealed in Begg’s test (*P* = 0.86) or Egger’s test (*P* = 0.40). The funnel plot appeared to be symmetrical (Fig. 8). For studies of the CV death outcome, the *P* value was 0.37 in Begg’s test and 0.07 in Egger’s test. The funnel plot was also symmetrical (Fig. 9).

## Discussion

The association of AF with the risk of thromboembolic stroke has been confirmed in previous studies; however, evidence regarding the association of AF with cardiac outcomes is lacking. A previous study suggested that a vast majority of deaths are related to cardiac causes (37.4%) rather than stroke (9.8%) in the contemporary anticoagulated AF population^57^. Although studies illustrated the association of AF with increased risk of SCD^58, 59^, it remains unclear whether AF is a predictor for SCD caused by coronary events. Besides, the meta-analysis revealed that AF is an independent predictor of mortality for patients with MI^11, 60^. However, the relation between AF and the development of MI remains poorly understood. Therefore, MI outcomes were studied, and the composite outcomes of CV death or events were selected as the main elements. This study was meaningful in summarizing and evaluating the current evidence of ischemic coronary outcomes in AF patients.

Pooled data from adjusted models showed a significant increase by 39% in the risk of MI for AF patients compared with non-AF individuals. This trend was insignificant when pooling crude RRs. It was inferred that potential confounding risk factors for MI might weaken the association of AF with MI. AF patients were associated with markedly increased likelihood of CV death or CV events. The risk of CV death or CV events in patients was approximately twice that in patients without AF (adjusted RR = 2.25 and crude RR = 1.95 for CV death; adjusted RR = 2.03 and crude RR = 2010 for CV events). A large long-term cohort showed no significant change in the trend of mortality among AF patients without a preexisting CV disease^61^, which supported the finding that AF is an independent predictor for CV death.

A previous study suggested an association of AF with increased risk of mortality in MI patients. New AF with no history of AF before MI remained associated with increased risk of mortality even after adjustment for several important risk factors for AF^62^. However, participants with other characteristics were not illustrated. Further, Emidin *et al*. conducted a meta-analysis of cohort studies to evaluate gender-related differences in the associations of AF with CV outcomes, indicating that AF is a stronger risk factor for cardiovascular disease and death in women compared with men, but the impact of confounders was not determined^63^. Important strengths of this meta-analysis include a comprehensive inclusion of relevant studies with a large sample size. Most cohorts were prospectively designed and population based. Long follow-up was found in most studies with a satisfying response rate. AF occurrence rates were reliably recorded following the International Classification of Diseases code or by direct evidence from electrocardiography (ECG) reports. Further, broad baseline characteristics that ensured the applicability of summary results to worldwide populations were included, representing global data of different regions or races. Both the adjusted and crude data were analyzed, which ensured completeness of the analysis, and demonstrated the potential impact of confounding factors. Overall, adjusted data were consistent in demonstrating the significant association of AF with studied outcomes. Crude data analysis failed to show significance only for MI. Also, data heterogeneity was assessed in multiple ways. No publication bias was found in the present study.

Several lines of mechanisms may explain the increased ischemic coronary burden for AF patients. AF could facilitate the induction of ventricular tachyarrhythmia, which is the predominant cause of SCD. This risk was especially high in patients with structural heart disease^64, 65^. In a porcine model, a rapid atrial pacing could induce ventricular ischemia and endothelial dysfunction in the microvasculature, and increased oxidative stress despite normal coronary vessels showing no atherosclerosis^66^. In AF patients, myocardial perfusion is impaired, with increased coronary vascular resistance. Notably, these changes are reversible to some extent after cardioversion of sinus rhythm^67^.

The impact of various AF patterns on different types of MI remains uncertain. Persistent AF results in worse patient survival compared with paroxysmal AF^68^. However, Senoo *et al*. suggested that the risk of cardiovascular death is higher in anticoagulated patients with permanent AF than in those with nonpermanent AF^69^. A large population-based study revealed that paroxysmal AF does not differ from other types of AFs in risk of overall mortality. In the adjustment model including concurrent MI, stroke, and HF, the risk of CV death is also independent of AF patterns^47^. Ruigómez *et al*. revealed that both chronic and paroxysmal AF cases are markedly associated with increased risk of coronary events, without a significant difference between the two subtypes^44^. A recent large cohort study revealed that AF is significantly associated with increased risk of non–ST-segment-elevation MI (NSTEMI) but not ST-segment-elevation MI (STEMI)^3^. It was inferred that partial occlusion of coronary arteries or increased oxygen demand, rather than coronary thrombo-embolization, is more likely to explain the observed association of AF with MI^3^.

Although adjusted data analysis showed significant results, various clinical or social factors might independently contribute to the development of ischemic coronary disease in AF patients, which deserves in-depth exploration^70–72^. The present findings may be confounded by differences in these measured or unmeasured baseline characteristics, including age, sex, race, hypertension, HF, smoking, and education. It has been demonstrated that black female AF patients, with warfarin prescription, and high CHADS2 score, are associated with higher risk of MI compared with their counterparts^51^. Of note, the roles of some relevant covariates remain controversial. The risk of acute myocardial infarction was shown to be higher in men with AF than in female counterparts^49^. However, the meta-analysis proved that AF was a stronger risk factor for CV events and death in women than in men^63^. With respect to antithrombotic therapy, different anticoagulation combinations, drug types, and treatment compliance are all possible confounding variables for CV outcomes. Some authors recommended adding antiplatelet drugs to vitamin K antagonist (VKA) when AF is complicated with coronary diseases. However, a national cohort revealed that addition of antiplatelet drugs to VKA therapy is not associated with decreased risk of coronary events in AF patients with coexisting coronary heart disease, HF, or vascular disease^73, 74^. The percentage of patients on antithrombotic therapy was low or unknown in the included cohorts. It is possible that many patients did not undergo standard anticoagulation following the guidelines^52^. Good anticoagulation was shown to be associated with a significant reduction in major cardiovascular events among AF patients^75^. Interestingly, a national cohort study suggested that future events tend to mirror the recent disease status in AF patients. For example, patients with previous MI have a higher risk of MI^76^. However, comorbid conditions were often not coded uniformly or described sufficiently among most cohorts.

Several limitations of this meta-analysis should be mentioned. Some studies identified AF patients by medical records rather than via ECG, and might have underestimated the number of undetected patients with asymptomatic AF at baseline^42^. Indeed, as an elusive arrhythmia, AF is difficult to ascertain in population-based cohorts. Data assessing the use of antithrombotic agents, AF type (paroxysmal, persistent, or chronic), and CHADS2 score were not collected in several large population-based cohorts^8, 33–36^. Meanwhile, inherent recall bias and selection bias were associated with retrospective cohorts^38, 45, 49^. Most studies did not collect information on continuous ECG recordings^49^. The information on percentage or indication for antithrombotic therapy could not be obtained in most cohorts. The majority of cohorts enrolled AF patients from individuals with heart disease or stroke^34, 36, 38, 39^, rather than healthy community-based enrollment, which might have limited the generalization of the current association with all AF populations. For CV death or events, categories may be heterogeneous among studies. Some studies may include stroke in these compound outcomes, which may exaggerate the association of AF with ischemic cardiac outcomes. Besides, the ascertainment of the cause of death may be liable to bias and misclassification^77^.

## Conclusions

In summary, AF was associated with MI, CV mortality, and CV events. The present study strongly supports the routine surveillance of ischemic heart disease, especially MI, among patients with AF. Future studies are warranted to clarify the interaction among confounding risk factors, and investigate effective preventive measures against adverse outcomes.

## Electronic supplementary material

Supplementary Tables S1-S3
