# Supplementary material for: Atrial fibrillation as a prognostic indicator of myocardial infarction and cardiovascular death: a systematic review and meta-analysis
Source: Sci Rep. 2017 Jun 13;7:3360. doi: 10.1038/s41598-017-03653-5 (PMC5469813; doi:10.1038/s41598-017-03653-5)
Supplement: Supplementary file 1 — Supplementary Tables S1-S3 [file 41598_2017_3653_MOESM1_ESM.doc]

**Atrial fibrillation as a prognostic indicator of myocardial infarction and cardiovascular death: a systematic review and meta-analysis**

Wenqi He^1^, Yingjie Chu^1*^

^1^Emergency department, Henan province People′s Hospital, Zhengzhou, Henan Province 450003, China

^1^This author takes responsibility for all aspects of the reliability and freedom from bias of the data presented and their discussed interpretation.

**E-mails for all authors:**

wenqi.he2008@163.com (Wenqi He)

HNQBDSL@126.com (Yingjie Chu)

**^*^Corresponding author:**

Yingjie Chu

Emergency department, Henan province People′s Hospital, Zhengzhou, Henan Province 450003, China

Tel: +86-18538236600

Fax: +86-21-64085875

E-mail: HNQBDSL@126.com

**Supplementary Table S1. Quality assessment of included studies using the modified Newcastle–Ottawa scale**

| Author (year) | Adequate definition  of cases with atrial fibrillation | Representativeness  of cases | Ascertainment of myocardial infarction or cardiovascular outcomes | Outcomes of interest were not present before atrial fibrillation | Control of confounding factors | Assessment  of outcomes | Adequate follow-up data (≥5 years) | Total score |
| --- | --- | --- | --- | --- | --- | --- | --- | --- |
| Kannel (1982) | 1 | 1 | 1 | 1 | 0 | 1 | 1 | 6 |
| Lake (1989) | 1 | 1 | 1 | 1 | 1 | 1 | 1 | 7 |
| Krahn (1995) | 1 | 1 | 1 | 1 | 1 | 1 | 1 | 7 |
| Aronow (1995) | 1 | 0 | 1 | 1 | 1 | 1 | 0 | 5 |
| Kaarisalo (1997) | 1 | 0 | 1 | 1 | 0 | 1 | 0 | 4 |
| Benjamin (1998) | 1 | 1 | 1 | 1 | 0 | 1 | 1 | 6 |
| Dries (1998) | 1 | 0 | 1 | 1 | 0 | 1 | 0 | 4 |
| Saxena (2001) | 1 | 0 | 1 | 1 | 0 | 1 | 0 | 4 |
| Friberg (2004) | 1 | 1 | 1 | 1 | 1 | 1 | 0 | 6 |
| Dhamoon (2007) | 1 | 0 | 1 | 1 | 1 | 1 | 0 | 5 |
| Goto (2008) | 1 | 0 | 1 | 1 | 0 | 1 | 0 | 4 |
| Ruigómez (2009) | 1 | 1 | 1 | 1 | 1 | 1 | 1 | 7 |
| Haywood (2009) | 1 | 0 | 1 | 1 | 0 | 1 | 0 | 4 |
| Bouzas-Mosquera (2010) | 1 | 0 | 1 | 1 | 1 | 1 | 1 | 6 |
| Winkel (2010) | 1 | 0 | 1 | 1 | 1 | 1 | 0 | 5 |
| Conen (2011) | 1 | 1 | 1 | 1 | 1 | 1 | 1 | 7 |
| Aguilar (2012) | 1 | 0 | 1 | 1 | 0 | 1 | 0 | 4 |
| Chao (2014) | 1 | 1 | 1 | 1 | 1 | 1 | 1 | 7 |
| Martinez (2014) | 1 | 0 | 1 | 1 | 0 | 1 | 0 | 4 |
| Soliman (2014) | 1 | 1 | 1 | 1 | 1 | 1 | 1 | 7 |
| Albayrak (2015) | 1 | 1 | 1 | 1 | 1 | 1 | 0 | 6 |
| Vermond (2015) | 1 | 1 | 1 | 1 | 1 | 1 | 1 | 7 |
| Soliman (2015) | 1 | 1 | 1 | 1 | 1 | 1 | 1 | 7 |
| Li (2015) | 1 | 1 | 1 | 1 | 1 | 1 | 0 | 6 |
| Parisi (2015) | 1 | 0 | 1 | 1 | 1 | 1 | 1 | 6 |
| Shih (2016) | 1 | 0 | 1 | 1 | 1 | 1 | 0 | 5 |
| O'Neal (2016) | 1 | 1 | 1 | 1 | 1 | 1 | 1 | 7 |

**Supplementary Table S2.** Subgroup analysis of crude relative risk for cardiovascular death and adjusted relative risk for cardiovascular death

| Outcomes | Group | RR and 95%CI | P value | Heterogeneity (%) | P value for heterogeneity | P value between subgroups |
| --- | --- | --- | --- | --- | --- | --- |
| **Crude relative risk for cardiovascular death** | Publication year | | | | | |
|  | 2010 or after | 2.21 (1.40-3.51) | 0.001 | 80.9 | 0.022 | 0.320 |
|  | Before 2010 | 2.27 (1.48-3.49) | <0.001 | 97.0 | <0.001 |  |
|  | Study design | | | | | |
|  | Prospective | 2.34 (1.56-3.49) | <0.001 | 96.7 | <0.001 | 0.646 |
|  | Retrospective | 1.82 (1.71-1.94) | <0.001 | - | - |  |
|  | Region | | | | | |
|  | North America | 2.81 (1.76-4.48) | <0.001 | 87.7 | <0.001 | <0.001 |
|  | Europe | 2.73 (0.54-13.78) | 0.224 | 99.4 | <0.001 |  |
|  | Asia | 1.23 (0.55-2.76) | 0.612 | 94.5 | <0.001 |  |
|  | International | 2.24 (1.95-2.56) | <0.001 | 0.0 | 0.853 |  |
|  | Sample size | | | | | |
|  | 10000 or greater | 2.79 (1.82-4.25) | <0.001 | 96.5 | <0.001 | <0.001 |
|  | < 10000 | 1.80 (1.19-2.73) | 0.005 | 93.6 | <0.001 |  |
|  | Mean age (years) | | | | | |
|  | 60 or older | 2.14 (1.58-2.89) | <0.001 | 95.5 | <0.001 | <0.001 |
|  | <60 | 2.91 (1.50-5.65) | 0.002 | 96.2 | <0.001 |  |
|  | Women proportion (%) | | | | | |
|  | ≥50 | 3.43 (1.87-6.29) | <0.001 | 96.9 | <0.001 | <0.001 |
|  | <50 | 1.54 (1.10-2.16) | 0.011 | 93.8 | <0.001 |  |
|  | Previous myocardial infarction (%) | | | | | |
|  | ≥20 | 1.96 (1.51-2.54) | <0.001 | 79.3 | 0.028 | <0.001 |
|  | <20 | 2.66 (1.40-5.05) | 0.003 | 97.3 | <0.001 |  |
|  | Adjustment degree | | | | | |
|  | +++ | 2.16 (1.30-3.59) | 0.003 | 97.1 | <0.001 | <0.001 |
|  | None | 2.36 (1.52-3.66) | <0.001 | 95.3 | <0.001 |  |
|  | Follow-up duration (years) | | | | | |
|  | ≥5 | 2.21 (1.33-3.67) | 0.002 | 91.6 | <0.001 | 0.518 |
|  | <5 | 2.32 (1.55-3.45) | <0.001 | 97.9 | <0.001 |  |
| **Adjusted relative risk for cardiovascular death** | Publication year | | | | | |
|  | 2010 or after | 1.94 (1.28-2.94) | 0.002 | 71.7 | 0.060 | 0.077 |
|  | Before 2010 | 1.95 (1.21-3.17) | 0.007 | 87.4 | <0.001 |  |
|  | Study design | | | | | |
|  | Prospective | 2.08 (1.40-3.07) | <0.001 | 82.5 | 0.001 | 0.020 |
|  | Retrospective | 1.65 (1.55-1.76) | <0.001 | - | - |  |
|  | Region | | | | | |
|  | North America | 1.81 (1.03-3.31) | 0.041 | 80.7 | 0.023 | <0.001 |
|  | Europe | 2.89 (2.23-3.74) | <0.001 | - | - |  |
|  | Asia | 1.65 (1.55-1.76) | <0.001 | 0.0 | 0.654 |  |
|  | Sample size | | | | | |
|  | 10000 or greater | 2.25 (1.46-3.48) | <0.001 | 90.0 | <0.001 | 0.231 |
|  | < 10000 | 1.51 (1.21-1.87) | <0.001 | 4.9 | 0.305 |  |
|  | Mean age (years) | | | | | |
|  | 60 or older | 1.65 (1.55-1.76) | <0.001 | - | - | 0.020 |
|  | <60 | 2.08 (1.40-3.07) | <0.001 | 82.5 | 0.001 |  |
|  | Women proportion (%) | | | | | |
|  | ≥50 | 2.25 (1.46-3.48) | <0.001 | 90.0 | <0.001 | 0.231 |
|  | <50 | 1.51 (1.21-1.87) | <0.001 | 4.9 | 0.305 |  |
|  | Previous myocardial infarction (%) | | | | | |
|  | ≥20 | 1.41 (1.11-1.80) | 0.005 | - | - | 0.066 |
|  | <20 | 2.05 (1.38-3.04) | <0.001 | 88.3 | <0.001 |  |
|  | Adjustment degree | | | | | |
|  | +++ | 1.95 (1.51-2.51) | <0.001 | 82.3 | <0.001 | - |
|  | ++ | - | - | - | - |  |
|  | Follow-up duration (years) | | | | | |
|  | ≥5 | 1.80 (1.27-2.55) | 0.001 | 63.2 | 0.066 | 0.748 |
|  | <5 | 2.15 (1.24-3.72) | 0.006 | 94.1 | <0.001 |  |

**Supplementary Table S3.** Subgroup analysis of crude relative risk for cardiovascular events and adjusted relative risk for cardiovascular events

| Outcomes | Group | RR and 95%CI | P value | Heterogeneity (%) | P value for heterogeneity | P value between subgroups |
| --- | --- | --- | --- | --- | --- | --- |
| **Crude relative risk for cardiovascular events** | Publication year | | | | | |
|  | 2010 or after | 2.17 (1.18-4.01) | 0.013 | 99.2 | <0.001 | <0.001 |
|  | Before 2010 | 1.81 (1.57-2.09) | <0.001 | 27.7 | 0.245 |  |
|  | Study design | | | | | |
|  | Prospective | 1.70 (1.46-1.98) | <0.001 | 67.0 | 0.010 | <0.001 |
|  | Retrospective | 2.75 (1.75-4.32) | <0.001 | 91.1 | 0.001 |  |
|  | Region | | | | | |
|  | North America | 1.61 (1.39-1.87) | <0.001 | 68.6 | 0.023 | <0.001 |
|  | Europe | 2.12 (1.75-2.57) | <0.001 | 0.0 | 0.983 |  |
|  | Asia | 3.40 (3.21-3.61) | <0.001 | - | - |  |
|  | Sample size | | | | | |
|  | 10000 or greater | 2.40 (1.17-4.90) | 0.016 | 95.5 | <0.001 | <0.001 |
|  | < 10000 | 1.81 (1.52-2.15) | <0.001 | 76.5 | 0.001 |  |
|  | Mean age (years) | | | | | |
|  | 60 or older | 1.85 (1.55-2.21) | <0.001 | 35.5 | 0.212 | <0.001 |
|  | <60 | 2.23 (0.99-5.01) | 0.052 | - | - |  |
|  | Women proportion (%) | | | | | |
|  | ≥50 | 1.75 (1.46-2.10) | <0.001 | 76.4 | 0.002 | <0.001 |
|  | <50 | 2.30 (1.40-3.78) | 0.001 | 93.6 | <0.001 |  |
|  | Previous myocardial infarction (%) | | | | | |
|  | ≥20 | 1.72 (1.55-1.92) | <0.001 | 0.0 | 0.397 | <0.001 |
|  | <20 | 2.23 (1.55-3.20) | <0.001 | 0.0 | 0.998 |  |
|  | Adjustment degree | | | | | |
|  | +++ | 2.17 (1.31-3.60) | 0.003 | 99.0 | <0.001 | <0.001 |
|  | ++ | 1.69 (1.49-1.91) | <0.001 | 0.0 | 0.496 |  |
|  | None | 1.64 (1.22-2.21) | 0.001 | - | - |  |
|  | Follow-up duration (years) | | | | | |
|  | ≥5 | 1.77 (1.24-2.52) | 0.001 | 79.1 | 0.008 | <0.001 |
|  | <5 | 2.14 (1.42-3.22) | <0.001 | 96.7 | <0.001 |  |
| **Adjusted relative risk for cardiovascular events** | Publication year | | | | | |
|  | 2010 or after | 2.16 (1.36-3.42) | 0.001 | 97.7 | <0.001 | 0.004 |
|  | Before 2010 | 2.13 (1.80-2.52) | <0.001 | 0.0 | 0.716 |  |
|  | Study design | | | | | |
|  | Prospective | 1.77 (1.52-2.07) | <0.001 | 55.8 | 0.035 | <0.001 |
|  | Retrospective | 2.77 (1.68-4.58) | <0.001 | 90.9 | 0.001 |  |
|  | Region | | | | | |
|  | North America | 1.91 (1.61-2.25) | <0.001 | 34.8 | 0.216 | <0.001 |
|  | Europe | 1.67 (1.41-1.98) | <0.001 | 35.0 | 0.202 |  |
|  | Asia | 4.12 (1.93-8.80) | <0.001 | 29.3 | 0.234 |  |
|  | Sample size | | | | | |
|  | 10000 or greater | 2.33 (1.04-5.22) | 0.040 | 99.3 | <0.001 | <0.001 |
|  | < 10000 | 1.90 (1.63-2.22) | <0.001 | 37.5 | 0.142 |  |
|  | Mean age (years) | | | | | |
|  | 60 or older | 1.83 (1.38-2.43) | <0.001 | 55.3 | 0.107 | <0.001 |
|  | <60 | 2.16 (1.10-4.23) | 0.024 | 61.8 | 0.073 |  |
|  | Women proportion (%) | | | | | |
|  | ≥50 | 1.87 (1.59-2.19) | <0.001 | 58.2 | 0.026 | <0.001 |
|  | <50 | 2.31 (0.99-5.38) | 0.052 | 96.6 | <0.001 |  |
|  | Previous myocardial infarction (%) | | | | | |
|  | ≥20 | 1.84 (1.24-2.73) | 0.003 | 77.1 | 0.037 | <0.001 |
|  | <20 | 1.90 (1.24-2.91) | 0.003 | 0.0 | 0.603 |  |
|  | Adjustment degree | | | | | |
|  | +++ | 2.08 (1.39-3.11) | <0.001 | 97.3 | <0.001 | 0.064 |
|  | ++ | 2.22 (1.79-2.75) | <0.001 | 0.0 | 0.982 |  |
|  | Follow-up duration (years) | | | | | |
|  | ≥5 | 1.72 (1.50-1.97) | <0.001 | 41.9 | 0.160 | <0.001 |
|  | <5 | 2.35 (1.52-3.64) | <0.001 | 92.0 | <0.001 |  |
